# Simultaneous expression of different transgenes in neurons and glia by combining *in utero* electroporation with the *Tol2* transposon-mediated gene transfer system

**DOI:** 10.1111/j.1365-2443.2010.01397.x

**Published:** 2010-05

**Authors:** Ayako Yoshida, Yoshifumi Yamaguchi, Keiko Nonomura, Koichi Kawakami, Yoshiko Takahashi, Masayuki Miura

**Affiliations:** 1 Department of Genetics, Graduate School of Pharmaceutical Sciences, The University of Tokyo7-3-1 Hongo, Bunkyo-ku, Tokyo 113-0033, Japan; 2 CREST, JST7-3-1 Hongo, Bunkyo-ku, Tokyo 113-0033, Japan; 3 Division of Molecular and Developmental Biology, National Institute of Genetics, and Department of Genetics, The Graduate University for Advanced Studies (SOKENDAI)1111 Yata, Mishima, Shizuoka 411-8540, Japan; 4 Graduate School of Biological Sciences, Nara Institute of Science and Technology8916-5, Takayama, Ikoma, Nara 630-0192, Japan

## Abstract

*In utero* electroporation is widely used to study neuronal development and function by introducing plasmid DNA into neural progenitors during embryogenesis. This is an effective and convenient method of introducing plasmid DNA into neural precursors and is suitable for manipulating gene expression in cells of the CNS. However, the applicability of this technique is comparatively limited to neuronal research, as the plasmid DNA introduced into neural progenitors during embryogenesis is diluted by cell proliferation and is not stably maintained in glial cells generated around and after birth. To overcome this limitation, we applied the *Tol2* transposon system, which integrates a transgene into the genome of the host cell, to *in utero* electroporation. With this system, we confirmed that the transgene was effectively maintained in the progeny of embryonic neural precursors, astrocytes and oligodendrocytes. Using the glial promoters GFAP and S100β, targeted and stable expressions of transgenes in glia were obtained, which enabled the expression of different transgenes simultaneously in neurons and glia. Glia-targeted expression of the transgene that causes neuronal migration defect was achieved without the defect. Thus, use of the *Tol2* transposon system in combination with *in utero* electroporation is a powerful method for studying glia-neuron interactions *in vivo*.

## Introduction

Evidence accumulated over the last decade has shown that glial cells not only support neuronal survival but also contribute significantly to the formation, operation and adaptation of neural networks (Wang & Bordey 2008). Although glia are often studied *in vitro*, *in vivo* investigations are required to elucidate their physiological roles. Currently, viral transduction and transgenic approaches are used to manipulate gene expression in glial cells *in vivo* (Levison & Goldman 1993; Burns *et al.* 2009). However, the surgery required to introduce genes of interest at postnatal stages causes local tissue damage and induces immune responses by microglia and astrocytes, which complicates the study of transgenes under normal, noninflammatory conditions. Although transgenic approaches are powerful and reliable strategies to study gene function, the financial costs limit their use. Thus, a more convenient and less invasive methodology is needed to accelerate the study of glial function by manipulating gene expression *in vivo*.

*In utero* electroporation is widely used to introduce transgenes into neural precursors to study neuronal development and function (Fukuchi-Shimogori & Grove 2001; Saito & Nakatsuji 2001; Tabata & Nakajima 2001). Compared with surgical viral transfer carried out at the postnatal stage, it is a convenient and less invasive method, similar to *in ovo* electroporation (Muramatsu *et al.* 1997; Funahashi *et al.* 1999). Typically, the *in utero* electroporation method involves injecting a conventional expression plasmid vector into the ventricles of the embryonic brain and using electrical pulses to transfer the DNA into the VZ (ventricular zone)/SVZ (subventricular zone) cells adjacent to the ventricles. The optimal developmental stage for using this method in mice is from about embryonic day (E) 10.5–16.5. Although this method is effective for studying neurons, as the plasmid is diluted at each division, it is not suitable to maintain the transgene in highly proliferative neural precursors and their descendants, glial cells (Miller & Gauthier 2007).

The *Tol2* transposon, from the medaka *Oryzias latipes*, has been used to integrate transgenes into the genomes of fish, amphibians, chicken and flies, as well as into mammalian cells in culture (Kawakami *et al.* 2000; Kawakami & Noda 2004; Hamlet *et al.* 2006; Tanabe *et al.* 2006; Sato *et al.* 2007; Watanabe *et al.* 2007; Urasaki *et al.* 2008). In the *Tol2* transposon–mediated gene transfer system (hereafter, the *Tol2* system), the *Tol2* transposase (T2TP) mediates integration of a transgene cassette flanked by *Tol2 cis*-sequences into the host genome. We reported using the *Tol2* system with the *in ovo* electroporation of chicken embryos, and achieving stable expression of the transgene in proliferative cells (Sato *et al.* 2007). These findings prompted us to combine the *Tol2* system with *in utero* electroporation to alter gene expression in glial cells in the mouse brain. We report here that our combination of transposon-mediated gene transfer system with *in utero* electroporation enables the expression of a transgene stably and efficiently in (i) mitotic neural precursor cells (radial glia) during development and (ii) macroglia, including both cortical astrocytes and oligodendrocytes, after birth. Moreover, this method allowed the labeling of neurons and glia with different markers and/or alteration of gene expression differentially and simultaneously in these cell types, using cell-type-specific promoters to drive different transgenes.

## Results

### Expression of a *Tol2*-flanked gene was maintained when introduced with the*Tol2* transposase

#### Expression in neural precursor cells (radial glia)

**Figure 1:**
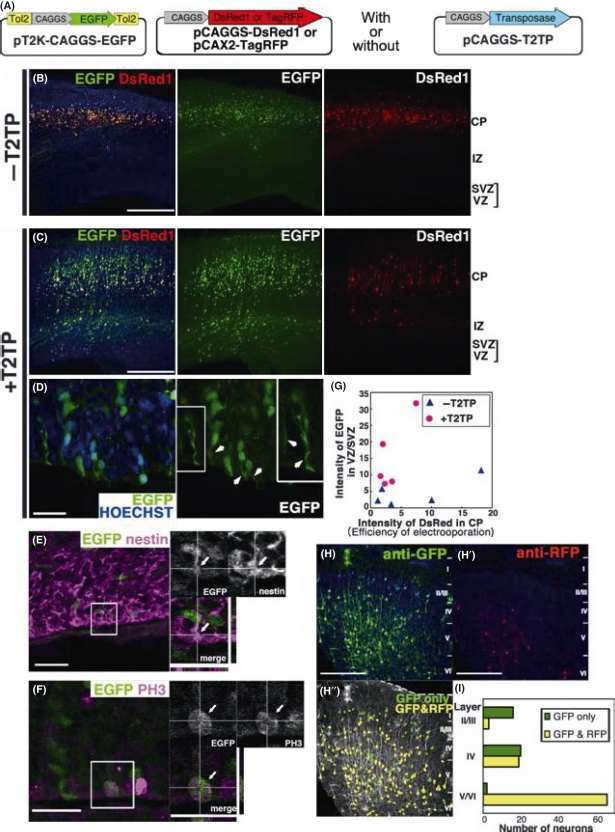
Persistent expression of *Tol2*-flanked EGFP in proliferative cells during neural development. (A) Schematic representation of plasmids introduced into the dorsolateral telencephalon of mouse embryos. (B–D) Coronal vibratome sections showing cerebral cortex electroporated at E11.5 and fixed at E16.5. (B) Without transposase. (C,D) With transposase. High-magnification view of VZ is shown in (D). Radial glia-like processes are indicated by arrows. (E,F) Expressions of EGFP introduced at E12.5 were observed after 4 days (E16.5) and some were positive for radial glial marker (nestin, E) or mitosis marker (PH3, F). Views from XY, XZ and YZ are shown in the right lower panels. (G) Relationship between the relative efficiency of electroporation and the retention of *Tol2*-flanked EGFP with or without T2TP. The relative efficiency of electroporation is represented as the relative intensity of DsRed1 in CP, and the retention of *Tol2*-flanked EGFP was assessed by evaluating the relative intensity of EGFP in the SVZ/VZ. (H,I) Retention of *Tol2*-flanked EGFP in late-born neurons. (H–H′′) Coronal cryosection of the cerebral cortex electroporated at E12.5 with T2TP and fixed at P8. The pial surface is indicated by the white dotted line. (I) The numbers of neurons expressing *Tol2*-flanked EGFP and/or TagRFP. Nucleus was counterstained with HOECHST 33342 (B–D,H). Scale bars, 250 μm in (B,C,H,H′) and 25 μm in (D–F).

We first investigated whether a transgene cloned into the *Tol2* vector was efficiently maintained in the progeny of electroporated neural progenitor cells during brain development. For this purpose, we introduced an expression cassette plasmid containing CAGGS-EGFP flanked by the *Tol2 cis*-sequences (pT2K-CAGGS-EGFP) with or without the plasmid for the *Tol2* transposase (pCAGGS-T2TP) into the mouse brain at E11.5 or E12.5 by *in utero* electroporation and examined EGFP expression in the brain at E16.5. pCAGGS-DsRed1, the plasmid containing CAGGS-DsRed1 without the *Tol2 cis*-sequences, was introduced along with the other plasmid(s) as a marker of electroporation efficiency (Fig. 1A). When pCAGGS-T2TP was not included, cells expressing EGFP and DsRed1 were both observed almost only in the cortical plate (Fig. 1B). In contrast, when pCAGGS-T2TP was included, broader EGFP expression was detected: it was expressed in the cortical plate (CP), intermediate zone (IZ), SVZ and VZ (Fig. 1C). Some of the EGFP-positive cells in the VZ had elongated radial glia-like processes extending to the edge of the ventricular zone (Fig. 1D, arrows) and were positive for the neural precursor marker, nestin (Fig. 1E). In addition, some of these precursors were located in the apical side, where mitosis occurs, and were positive for the mitosis marker, phospho-Histone H3 (PH3) (Fig. 1F). The intensity of EGFP fluorescence in the T2TP-transduced VZ/SVZ was much higher than that in the same regions without T2TP introduction, regardless of the electroporation efficiency (Fig. 1G). The effect of T2TP was not dependent on which fluorescent proteins were flanked by the *Tol2 cis*-sequences (data not shown).

Next, we examined whether the *Tol2*-flanked transgene was inherited by the descendants of the electroporated radial glia. We carried out *in utero* electroporation with pT2K-CAGGS-EGFP, pCAGGS-T2TP and the control plasmid, pCAX2-TagRFP, at E12.5 and analyzed the distribution of neurons expressing EGFP and TagRFP on postnatal day (P) 8, when radial neuronal migration is mostly finished. We anticipated that EGFP expression would overlap with TagRFP expression in the inner cortical layers but would extend farther than TagRFP in the outer layers, because neurons of the outer cerebral cortical layers are born later than those in the inner layers (Takahashi *et al.* 1999). Indeed, neurons expressing EGFP were found from layer VI to upper layer II/III, whereas those expressing TagRFP were confined mainly to layers VI to IV (Fig. 1H,I). These results suggested that the *Tol2* transposase stably integrated the *Tol2*-flanked transgene into the genome of proliferative neural progenitors.

#### Expression in perinatally generated glial cells

**Figure 2:**
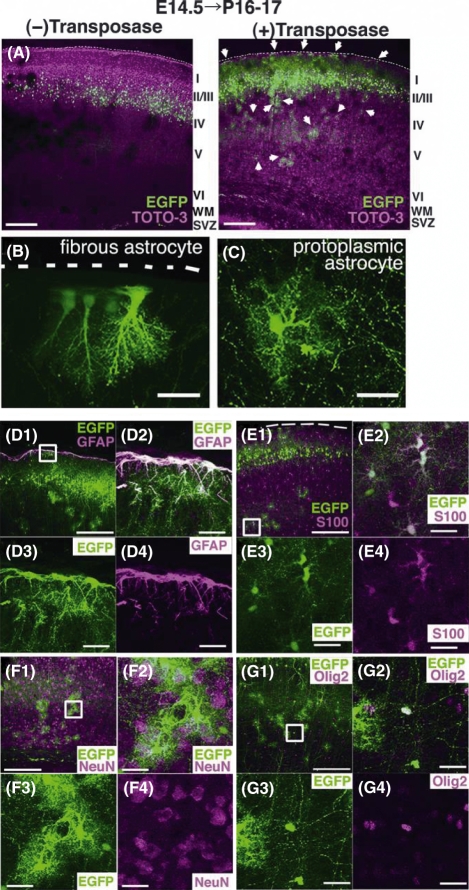
*Tol2*-flanked transgene was inherited by glial cells generated postnatally. (A) Emergence of non-neuronal cells labeled by *Tol2*-flanked EGFP (right, arrows). The dorsal telencephalon was electroporated to introduce pT2K-CAGGS-EGFP at E14.5 without (left) or with (right) pCAGGS-T2TP. The brain was analyzed at P16–17. (B,C) Higher magnification of EGFP-positive cells with non-neuronal morphology showing fibrous astrocytes in the marginal zone (B) and protoplasmic astrocytes in the cortical plate (C). (D–G) Immunostaining of vibratome sections against cell-type-specific markers. Higher magnification view of the insets in 1 is shown in 2–4. EGFP-expressing cells were positive for GFAP (D), S100 (E), or Olig2 (G) and negative for the neuronal marker, NeuN (F). Merged image of EGFP and cell type markers is shown in 2. Scale bars, 250 μm (A,D1,E1,F1,G1) and 25 μm (B,C,D2–4,E2–4, F2–4,G2–4). The pial surface is indicated by the white dotted line.

As neural progenitor cells switch from generating neurons to generating glia around the time of birth, we next examined whether glial cells could inherit the transgene introduced into the electroporated neural progenitor cells using our system. pT2K-CAGGS-EGFP was introduced into the cortex by *in utero* electroporation at E14.5 with or without pCAGGS-T2TP, and the expression of EGFP was examined at the stages when glial morphological differentiation was evident (P16–17). As shown earlier, when T2TP was not expressed, EGFP was found only in pyramidal neurons located mainly in layers II–IV, which are born just after E14.5 (Fig. 2A, left panel) (Langevin *et al.* 2007). When T2TP was co-introduced with pT2K-CAGGS-EGFP, EGFP was expressed not only in the pyramidal neurons of the cortical plate, but also in cells showing morphologies and distributions distinct from those of neurons (Fig. 2A, right panel, and B,C). These non-neuronal EGFP-positive cells were observed from the SVZ to the marginal zone of the cortex (Fig. 2A right, arrows). The majority exhibited features characteristic of two types of astrocyte: fibrous astrocytes, which are located mainly in layer I and white matter and have a root-like morphology (Fig. 2B), and protoplasmic astrocytes, which are distributed throughout the cortex and have a bushy shape (Fig. 2C) (Kimelberg 2004).

We also confirmed the astrocytic properties of these EGFP-positive cells by immunohistochemical staining for GFAP (Fig. 2D) and for S100 (Fig. 2E). Neither of these cell types showed labeling with an antibody against NeuN, a neuronal marker (Fig. 2F). Some of the non-neuronal EGFP-positive cells were also positive for Olig2 (Fig. 2G), indicating that the stable integration of the transgene into neural progenitors also led to transgene expression in both astrocyte and oligodendrocyte lineages (Levison & Goldman 1993; Ono *et al.* 2008).

**Figure 3:**
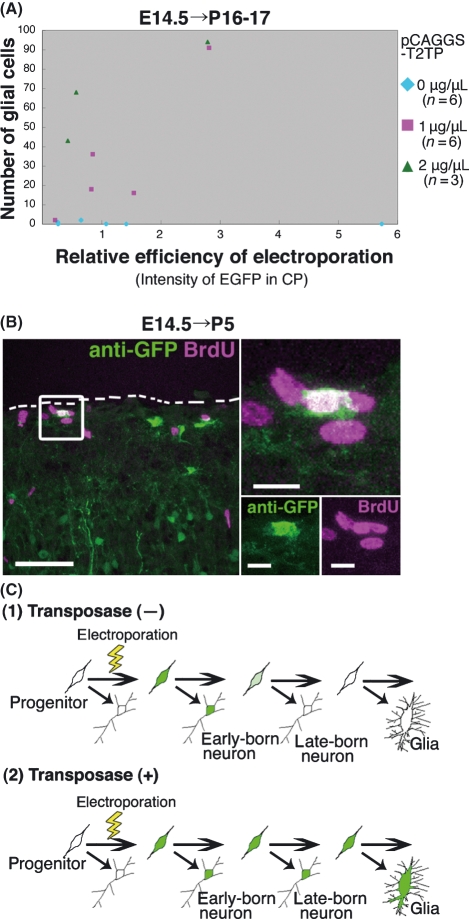
The *Tol2* system resulted in postnatal expression of the transgene by mitotic cells. (A) Relationship between the relative efficiency of electroporation and the number of labeled glial cells at P16–17. (B) Retention of the transgene by mitotic cells at P5 after *in utero* electroporation at E14.5. Some EGFP-positive cells (green) were also positive for BrdU (magenta). A higher magnification view of the inset is shown. Scale bars, 50 μm (left panel) and 10 μm (right three panels). The pial surface is indicated by the white dotted line. (C) Schematic image of the *Tol2* transposon system combined with the *in utero* electroporation method. (1) Without transposase, the plasmid introduced into progenitor cells was diluted following cell proliferation, and the expression of the transgene was restricted to cells born and becoming postmitotic soon after electroporation. (2) With the *Tol2* transposase, a transgene flanked by the *Tol2 cis*-sequences was integrated into the genomic DNA of progenitor cells and inherited by its descendants, including neurons and glia.

EGFP expression in glial cells was almost completely dependent on T2TP co-electroporation (Fig. 3A). To verify that the electroporated cells were proliferative postnatally, we carried out BrdU labeling at the stage when vigorous gliogenesis occurs (P4) and observed the mitosis of EGFP-positive cells (Fig. 3B). Thus, combining *in utero* electroporation with the *Tol2* system enabled the expression of a transgene in glial cells born after neurogenesis was completed (Fig. 3C).

### Glia-targeted gene expression using cell-type-specific promoters

Although our method allowed the expression of various transgenes in late-born glia using the broadly active CAG promoter with the *Tol2* system, the persistent and strong expression of a transgene from the neurogenic stages to postnatal life may affect neuronal development, in turn leading to aberrant glial development. To dissect the molecular mechanisms that underlie the development and physiological function of glial cells, glia-targeted manipulation of various genes is required. Therefore, we used two cell-type-specific promoters, i.e. those for mouse GFAP (Miura *et al.* 1990) and S100β (Vives *et al.* 2003), which are expressed primarily in glial cells, mainly in astrocytes.

**Figure 4:**
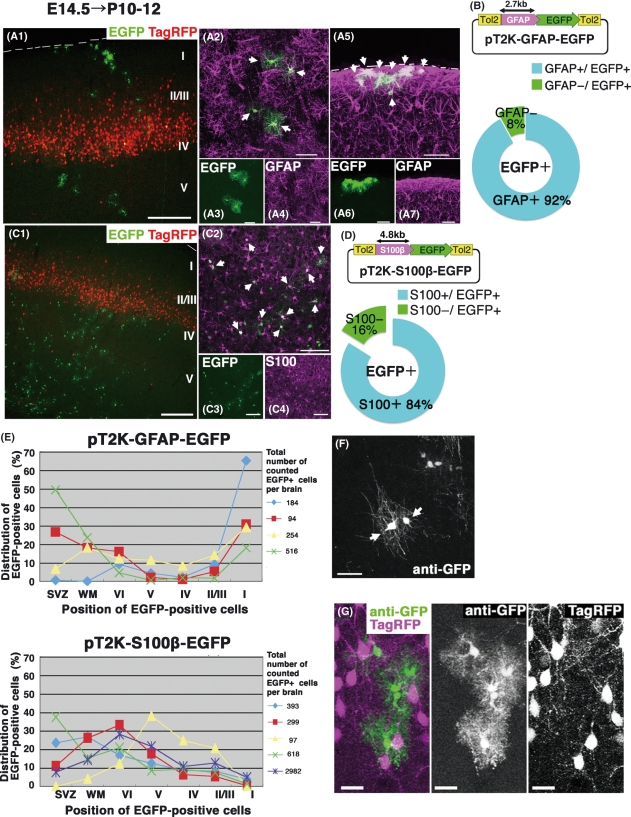
The mouse GFAP promoter and S100β promoter drove targeted expression of the transgene in glial cells. (A–G) Astrocyte-targeted expression of EGFP driven by the mouse GFAP promoter or S100β promoter. The E14.5 telencephalon was electroporated to introduce pT2K-GFAP-EGFP (A,B) or pT2K-S100β-EGFP (C,D) along with pCAX2-TagRFP and pCAGGS-T2TP and analyzed at P10–12. TagRFP was expressed in pyramidal neurons (A,C). Higher magnification views of double-labeled cells with EGFP by each promoter and cell type marker are shown in (A2–7) (GFAP) or (C2–4) (S100) (white arrows). The pial surface is indicated by the white dotted line. (B,D) Percentages of GFAP- or S100-positive cells among EGFP-positive cells. (B: 124 cells/4 brains, D: 632 cells/5 brains) (E) Distribution of pT2K-GFAP-EGFP- or pT2K-S100β-EGFP-positive cells throughout the cortex. Vertical lines indicate the ratio of EGFP-positive cells in each layer against total EGFP-positive cells in the brain. Each line indicates an individual brain (GFAP; *n*=4, S100β; *n*=5). (F) Cells exhibiting typical morphology of oligodendrocytes. (G) Dual differential labeling of astrocytes and neurons by pT2K-S100β-EGFP and pCAX2-TagRFP, respectively. Scale bars, 250 μm (A,C, left), 50 μm (A, right six panels, F), 100 μm (C, right three panels) and 25 μm (G).

*In utero* electroporation of a plasmid encoding *Tol2*-flanked EGFP driven by the mouse GFAP promoter (pT2K-GFAP-EGFP) and pCAGGS-T2TP at E14.5 into lateral VZ of the neocortex resulted in EGFP expression restricted to astrocytes at the stages when astrocytes are distributed throughout the cortex (P10–12) (Fig. 4A). In these experiments, neurons were labeled with TagRFP, encoded by the pCAX2-TagRFP plasmid, which was co-introduced during electroporation and was not flanked by the *Tol2 cis*-sequences. Almost all of the EGFP-positive cells (92%) also expressed the endogenous GFAP protein (Fig. 4B). Similarly, co-electroporation of the S100β promoter-driven EGFP (pT2K-S100β-EGFP) with pCAGGS-T2TP at E14.5 caused EGFP expression in astrocytes distributed throughout the cortex at P10–12 (Fig. 4C). Approximately 84% of the EGFP-positive cells showed colocalized immunoreactivity for the S100 protein (Fig. 4D). In both experiments, the expression patterns of EGFP were evaluated by counting cell numbers in each layer (layer I–VI, white matter: WM, and SVZ). Cells expressing pT2K-GFAP-EGFP tended to localize in layer I, WM and SVZ, whereas those expressing pT2K-S100β-EGFP were distributed throughout the cortex (Fig. 4E). Taken together, our data show that glial-targeted expression of an exogenous gene using cell-type-specific promoters was achieved. In addition, immunostaining against GFP visualized cells exhibiting morphology typical of oligodendrocytes, suggesting that the *Tol2* transposase enables introduction of transgenes into both astrocyte and oligodendrocyte lineages by *in utero* electroporation (Fig. 4F and see also Fig. 2G) (Zerlin *et al.* 2004). Furthermore, these experiments demonstrated that two different transgenes could be expressed in astrocytes and adjacent neurons simultaneously and independently (Fig. 4G).

**Figure 5:**
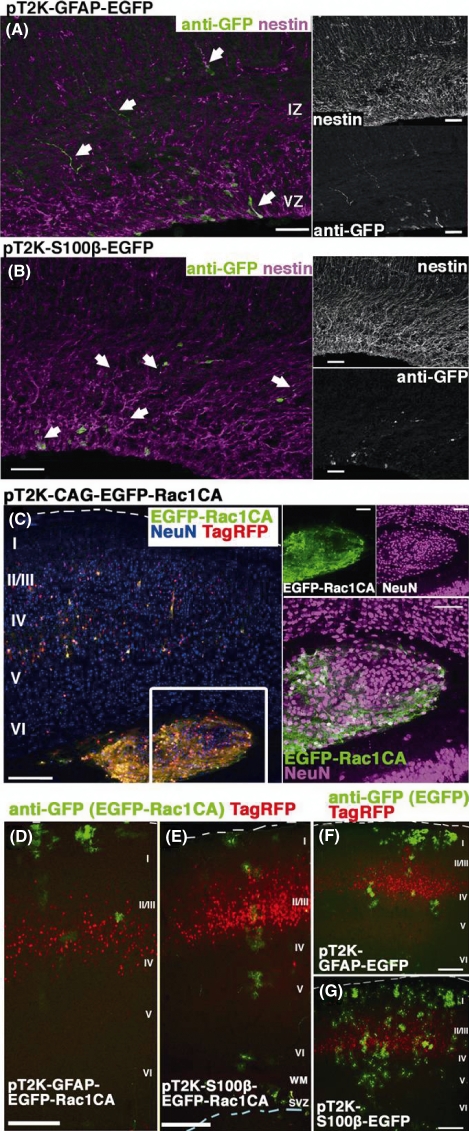
Targeted expression of constitutively active Rac1 (Rac1CA) under the control of the mouse GFAP or S100β promoter did not affect neuronal migration. (A,B) Expression of pT2K-GFAP- or pT2K-S100β-EGFP detected by anti-GFP immunostaining at E18.5. Some cells expressing EGFP were positive for the radial glial marker, nestin (arrows). (C–G) Distributions of cells expressing pT2K-CAG-EGFP-Rac1CA (C), pT2K-GFAP-EGFP-Rac1CA (D), pT2K-S100β-EGFP-Rac1CA (E), pT2K-GFAP-EGFP (F) or pT2K-S100β-EGFP (G). Glial-targeted expressions were visualized by anti-GFP immunostaining (D–G). Lateral neocortex electroporated at E14.5 was examined at P16 (C) or P10 (D–G). Scale bars, 50 μm (A,B), 250 μm (C, left panel, D–G) and 100 μm (C, three rightmost panels). The pial surface or the border between the ventricle and SVZ is indicated by a white or cyan dotted line, respectively.

We next examined whether these glia-targeted promoters with the *Tol2* system could be used to express a transgene without affecting neuronal development. As it is known that radial glial cells begin to express GFAP at later stages of neurogenesis, we examined the expression of pT2K-GFAP- or pT2K-S100β-EGFP at E18.5, 4 days after electroporation. Both pT2K-GFAP-EGFP and pT2K-S100β-EGFP expressions were detected in nestin-positive radial glia by immunostaining with anti-GFP antibody (Fig. 5A,B), although the level of EGFP expression was too low for visualization without staining (data not shown). Previous studies showed that transient expression of dominant negative or constitutively active form of Rac1 (Rac1CA) under the CAG promoter disturbed the radial migration of neurons (Kawauchi *et al.* 2003; Konno *et al.* 2005). We confirmed that when we used our system to express Rac1CA under the control of the CAG promoter, only a few neurons expressing the exogenous gene were present in the cortical plate even at P16, 1 week after completion of neuronal migration; most remained in the SVZ, consistent with the results obtained by transient expression (Fig. 5C) (Konno *et al.* 2005). In contrast, when we expressed Rac1CA under the control of the mouse GFAP or S100β promoter, neurons expressing the co-electroporated pCAX2-TagRFP were located normally in layer II–IV at the stage when neuronal migration has almost finished (P10) (Fig. 5D,E). Astrocytes expressing Rac1CA were also observed throughout the cortex. However, the number of labeled astrocytes was reduced, compared with the control (Fig. 5D–G), suggesting that the expression of Rac1CA may affect early gliogenesis or survival. These results indicated that using a glia-targeted promoter with *Tol2* transposon system facilitates investigation into the function of genes involved in glial development and physiology without affecting neuronal development.

## Discussion

*In utero* electroporation was first reported in 2001 and has become widely used in the fields of developmental biology and neuroscience (Fukuchi-Shimogori & Grove 2001; Saito & Nakatsuji 2001; Tabata & Nakajima 2001). However, its application to date has been limited to neurons. Use of high plasmid concentrations makes it possible to maintain and express transgenes for several days in proliferative radial glia and therefore is useful for studying neurogenesis. With regard to gliogenesis, it was reported that conventional *in utero* electroporation using higher plasmid concentrations or carried out at a later stage resulted in glial transgene expression (Tabata & Nakajima 2001; LoTurco *et al.* 2009). However, these strategies could not always lead to stable inheritance in mitotic cells in a reproducible manner, and therefore, they were not suitable for glial study at postnatal stages. In fact, there have been no reports of the use of conventional *in utero* electroporation for postnatal glial studies. Furthermore, peri/postnatal electroporation or virus transfection may result in tissue damage and inflammation (Buffo *et al.* 2008; Silver & Steindler 2009). In contrast, combining *in utero* electroporation with the *Tol2*-mediated gene transfer system allows the introduction of transgenes when the immune system is immature, and the plasticity of developing tissues affords perturbation. This is a great advantage for studying glia, which is sensitive to environmental changes, both during postnatal development and in physiological contexts. This ‘preloading of transgenes’*in utero* has been carried out by retrovirus infection (Walsh & Cepko 1988; Noctor *et al.* 2001; Stott & Kirik 2006). The retroviral technique is very useful for lineage tracing analysis by visualizing individual cells during development, although it requires the time-consuming process of packaging retrovirus and strict control of the virus titer. In contrast, the *Tol2* system is a convenient method for integrating conventional and multiple plasmid expression vectors into the host cell genome to achieve stable expression. These features will facilitate investigation into the role of various genes in glial cells.

Previous studies showed that the neuron-specific Tα1 promoter and radial glia-specific nestin promoter are useful for labeling neuronal subtypes during development by *in utero* electroporation (Tabata & Nakajima 2001; Gal *et al.* 2006; Wang *et al.* 2007). Similarly, we showed here that astrocyte-targeted expression can be achieved by *in utero* electroporation using the mouse GFAP and S100β promoters combined with the *Tol2* system. Although expressions of both the promoters were observed in radial glial cells at E18.5, we could express EGFP-Rac1CA specifically under the control of promoters in glia without perturbing neuronal migration. Although further studies are required to determine whether the mechanism of migration is different between astrocytes and neurons or overexpression of Rac1CA is cytotoxic for gliogenesis and/or astrocytes, our results strongly suggest that the *Tol2*-mediated gene transfer system is very useful for investigating molecular/cellular function of genes of interest in glial development and physiology without affecting neurons.

The number of EGFP-expressing astrocytes was dependent on the efficiency of electroporation and the amount of T2TP plasmid, as shown in Fig. 3A. This suggests that it is possible to modulate labeling efficiency to some extent by altering the volume or concentration of plasmid injected into the ventricle. However, inherent to this type of experimental manipulation, it was difficult to control the number of labeled cells precisely. At best, more than 400 cells per 150-μm slice of P10 brains could be successfully and reproducibly induced to express EGFP driven by the S100β promoter (data not shown). This level of transgene expression should be more than sufficient for examining the behavior of single glial cells or for detecting biological processes with a reporter gene *in vivo*. In this respect, this system should be suitable for studying glial–neuronal interactions *in vivo*, because different transgenes can be expressed in a mutually exclusive manner in neurons and glia, greatly facilitating such investigations (Fig. 4G). Likewise, it will permit a variety of investigations regarding glial development, glial–blood vessel interactions and glial responses to environmental challenge. These usages can be extended by adding RNAi experiments to the repertoire, as the *Tol2* system is compatible with RNAi use, at least in chicken embryos (Watanabe *et al.* 2007). Recent exhaustive studies using microarray or proteomics analyses have identified many genes that are expressed in a cell-type-specific manner. To screen candidate genes for important roles in glial cells, using the *Tol2*-mediated gene transfer system *in vivo* would allow the manipulation of gene expression with less risk of infection or of triggering immune responses. Therefore, this method may be used with gene profiling methodologies, such as the TRAP system by mRNA labeling of glial cells *in vivo* (Doyle *et al.* 2008; Heiman *et al.* 2008).

In conclusion, this *Tol2*-mediated gene transfer system combined with *in utero* electroporation is a powerful new option for glial study in addition to viral transfection and transgenic strategies. It will allow the analysis of numerous aspects of astrocytic development and function and to dissect the contributions of the responsible genes both conveniently and effectively.

## Experimental procedures

### Vector plasmids

The constructs pT2K-CAGGS-EGFP, pT2K-BI-EGFP, pCAGGS-T2TP and pCAGGS-DsRed1 were previously reported (Sato *et al.* 2007). To make pT2K-GFAP-EGFP, pT2K-BI-EGFP was digested with *Eco*RI to remove the cassette containing the TRE sequence. This site was blunted, and a blunted DNA fragment containing the mouse GFAP promoter, which was prepared from pGF1L (Miura *et al.* 1990) with *Hin*dIII, was inserted. To make pT2K-S100β-EGFP, a 4.8-kb mouse genomic DNA sequence containing the S100β promoter region was amplified by PCR as described previously (Vives *et al.* 2003). The PCR product was inserted into *Nde*I-*Eco*RI site of pT2K-BI-EGFP. pT2K-GFAP-EGFP-Rac1CA and pT2K-S100β-EGFP-Rac1CA were based on pT2K-GFAP-EGFP, which was digested with *Nco*I, or pT2K-S100β-EGFP, digested with *Eco*RI, blunt-ended, and digested with *Cla*I. The vectors were ligated with a DNA fragment of pEGFP-Rac1V12 (Yasuda *et al.* 2004), amplified by PCR (primers: 5′;CATGCCATGGTGAGCAAGGGCGAGG, 3′;CCATCGATCCTTACAACAGCAGGC), and digested with *Cla*I. To make pT2K-CAG-EGFP-Rac1CA, pT2K-CAGGS-EGFP digested with *Xba*I & *Eco*RV was ligated with the DNA fragment of pEGFP-Rac1V12 treated with *Nhe*I & *Hpa*I. pCAX2-TagRFP was made from pCAX2, which contains the CAG promoter (an upstream cytomegalovirus enhancer and chicken β-actin promoter sequence). The pCAX2 was digested with *Eco*RI and *Not*I and was ligated with a DNA fragment of pTagRFP-N (#FP142; Evrogen), obtained by digestion with *Eco*RI and *Not*I.

### Animals, electroporation and tissue processing

For the *in utero* electroporation, we used timed pregnant ICR females at the indicated day of gestation. Mice were anesthetized with sodium pentobarbitone at 70 μg per gram of body weight. The surgery and manipulation were carried out as described (Tabata & Nakajima 2001) with the modifications as following. Plasmid concentration: 0.5 to 3 μg per μL, Electrode: CUY650P3 or CUY650P5 (Nepa Gene) for E11.5, E12.5 or E14.5, respectively, Electronic pulses: 33 V, four times, 30 or 50 ms at intervals of 970 or 950 ms at E11.5, E12.5 or E14.5, respectively. The uterus was gently replaced, the incision was closed, and normal development was allowed to continue. Mice were killed at time points between E16.5 and P17 by cervical dislocation or perfusion fixation with 2–4% PFA, and the brain was removed. Each brain was immersed in 4% PFA for 60–120 min, embedded in 2% low melting point agar (NuSieve® 3 : 1 Agarose; Lonza) and sliced into 150–180 μm coronal sections on a vibratome (VT1200S; Leica). To analyze the expression of transgene in radial glia at E16.5 or 18.5 or the distribution of neurons at P8 and to evaluate BrdU incorporation, the fixed brains were sunk in 25% sucrose and subsequently embedded in O.C.T. compound (Sakura) and frozen in liquid nitrogen. Frozen coronal sections (20–30 μm) were cut on a cryostat (CM3050S; Leica) (Table 1).

**Table 1:** Concentration of plasmids introduced by *in utero* electroporation (μg/μL)

	CAG-EGFP at E11.5	CAG-EGFP at E12.5	CAG-EGFP at E14.5	Birth-date labeling	GFAP/S100β-EGFP	CAG-EGFP-Rac1CA	GFAP/S100β-EGFP-Rac1CA
pT2K-CAGGS-EGFP	0.6	0.6	3.0	3.0			
pT2K-GFAP/S100β-EGFP					1.5		
pT2K-CAG-EGFP-Rac1CA						2.0	
pT2K-GFAP/S100β-EGFP-Rac1CA							1.5
pCAGGS-T2TP	1.5	1.5	0–2.0	1.0	1.0	1.0	1.0
pCAX2-TagRFP		0 or 1.5			0.5–1.0	1.0	0.5–0.75
pCAGGS-DsRed	1.5		1.5				
Electroporation→Fixation	E11.5→E16.5	E12.5→E16.5 or P8	E14.5→P16–17	E14.5→P5 (BrdUinjectionat P4)	E14.5→E18.5 orP10–P12	E14.5→P16	E14.5→P10

### Immunohistochemistry

The primary antibodies were polyclonal rabbit anti-tRFP Ab (AB232, 1 : 1000 dilution; Evrogen), mouse monoclonal anti-GFP Abs (#11814 460001, 1 : 500 or 1 : 1000; Roche), rabbit polyclonal anti-GFP Ab (#598, 1 : 1000; MBL), rabbit polyclonal anti-PH3 (06-570, 1 : 500; Upstate), mouse monoclonal anti-nestin (Rat401; Developmental Studies Hybridoma Bank, University of Iowa; 1 : 50), mouse monoclonal anti-GFAP (G3893, 1 : 1000; Sigma), mouse monoclonal anti-NeuN (MAB377, 1 : 1000; Chemicon), rabbit serum anti-Olig2 (a kind gift from H. Takebayashi; 1 : 1000), rabbit polyclonal anti-S100 antibody (Z0311, 1 : 500; Dako), rat monoclonal anti-BrdU Ab (ab6326, 1 : 500; Abcam) and rat monoclonal anti-GFP Ab (#04404-26, 1 : 1000; Nacalai-tesque). Sections cut on a Vibratome or cryostat were soaked in blocking buffer (20% immunoblock; DS Pharma Biomedical Co., Ltd.) in PBST (0.1% Triton-X in PBS) for 30–60 min and incubated with primary antibodies in PBST overnight (or up to several days) at 4 °C. After several washes in PBST, slices were incubated with fluorescence-labeled secondary antibodies (Jackson Immunoresearch Lab) overnight at 4 °C. All sections were costained with TOTO-3 iodide (T3604, 1 : 3000; Invitrogen) or HOECHST33342 (2 μm) to label nuclei. Pictures were acquired with a TCS SP5 confocal microscope (Leica).

### Quantitative analyses

The relative efficiency of electroporation in E16.5 animals (Fig. 1G) was estimated by the relative intensity of DsRed1 in the CP on cryostat sections. Fluorescence intensity was measured with LAS AF software (Leica) on the maximum projection of intensity on images of a confocal z-stack. The effect of T2TP on the retention of a transgene was determined by measuring the intensity of EGFP in the SVZ/VZ on the same sections. The CP and SVZ/VZ were defined by morphological criteria.

In animals killed postnatally (Fig. 3A), the relative efficiency of electroporation (I) was estimated by the intensity of EGFP in the CP on vibratome brain slices. The fluorescence intensity of each slice was measured by Image J from its picture taken on a camera-equipped fluorescence stereoscopic microscope (Leica). For each brain, the numbers of labeled glial cells were counted in one slice; the slice that had the highest EGFP intensity in CP was used. The slices were observed on a confocal microscope, and cells with a bushy morphology (clearly distinct from that of neurons) were counted as glial cells. Because the thickness of the sections and area scanned by confocal microscopy to count glial cells differed among the samples, we normalized the glial cell number (G) by dividing it by the volume (V) [slice thickness (μm) × the area]. To represent the relationship between (G) and (I), (G) was plotted against (I × V), which is equal to (G/V) against (I).

For analyzing expression pattern of EGFP driven by GFAP or S100β promoter, EGFP-positive cells were taken pictures with confocal microscopy on all sections and their numbers were counted. These numbers were classified by their location into cerebral cortex, SVZ, WM, and from layer VI to I. The expression patterns in whole brain were obtained as a total of sections per individual and plotted in the graph.

### BrdU labeling

Newborn mice subjected to *in utero* electroporation at E14.5 were given an injection of BrdU of 100 μg/g body weight at P4 and killed 28 hours later. Frozen sections were prepared from each brain as described earlier and incubated with rabbit polyclonal anti-GFP Ab (1 : 1000) for 3 hours at room temperature (rt) and then with secondary antibodies for 1 hour at rt. The sections were fixed again with 4% PFA for 20 min at rt, washed in PBS twice, incubated with 2 N HCl for 15 min at 37 °C, washed in PBS twice, incubated with blocking buffer for 30 min at 4 °C and then incubated with rat monoclonal anti-BrdU Ab overnight at 4 °C. After three washes in PBST, they were incubated with secondary antibody for 1 hour at rt and mounted.
